# Age-specific prostate specific antigen and prostate specific antigen density values in a community-based Indian population

**DOI:** 10.4103/0970-1591.32060

**Published:** 2007

**Authors:** Arvind P. Ganpule, Mahesh R. Desai, T. Manohar, Sharad Bapat

**Affiliations:** Department of Urology, Muljibhai Patel Urological Hospital, Dr. V. V. Desai Road, Nadiad, Gujarat, India

**Keywords:** Community study, prostate specific antigen density ranges

## Abstract

**Introduction:**

We sought to determine the distribution of serum prostate specific antigen (PSA) levels in a community-based population in the state of Gujarat in India.

**Materials and Methods:**

Community screening of subjects residing in Kheda and Anand districts of Gujarat was done with digital rectal examination (DRE), uroflowmetry and transabdominal sonography and serum PSA. In three villages transrectal sonography was done in addition to transabdominal sonography. All patients with a PSA of more than 4 ng/ml and / or abnormal DRE were evaluated. Biopsy-proven prostatic malignancy was detected in 20 patients. Subjects who did not follow up and who had biopsy-proven malignancy were excluded from the analysis, 1899 subjects were analyzed.

**Results:**

One thousand seven hundred and eighty-seven (89.17%) had a PSA of <4 ngml, 180 (8.9%) had a PSA between 4-10 ng/ml, while 37 (1.8 %) had a PSA more than 10ng/ml. There was a statistically significant correlation between age and prostate volume (correlation coefficient 0.33) and between prostate volume and PSA (correlation coefficient 0.50). The age-specific PSA values derived as the 95^th^ percentile value were as follows, 40-49 years (0-2.1), 50-59 years (0-3.4), 60-69 years (0-4.2) and more than 70 years (0-5.0). The 95^th^ percentile value for PSAD was 0.19.

**Conclusion:**

Indians need to have separate PSA reference ranges. The age-specific PSA ranges for men in the present study population are on the lower side and the prostate specific antigen density on the higher side.

Prostate specific antigen (PSA) has been described as a single test with highest positive predictive value for diagnosing carcinoma prostate.[1] Routine use of PSA helps in diagnosing organ-confined prostate cancers.

The PSA thresholds such as age-specific PSA, PSA density (PSAD) and PSA velocity improve the specificity and sensitivity of PSA. Osterling *et al* observed that serum PSA, prostate volume and PSA density values have racial differences. They noted that community-based Japanese population had different PSA values and prostate volume as compared to the American population.[2]

Indians being ethnically distinct from Caucasians and Japanese, we sought to determine the distribution of serum PSA levels in a community-based population in the state of Gujarat in India.

## MATERIALS AND METHODS

We conducted a community screening of subjects residing in the Kheda and Anand districts of Gujarat. Approval from the ethical committee, Muljibhai Patel Urological hospital was taken for carrying out the screening. The population was informed through electronic and print media regarding the date and venue of community screening, the help of local nongovernment organizations was also taken. We also distributed pamphlets informing the subjects about the nature of screening and who may benefit from it.The screening at the venue started with an in-depth lecture on the purpose and objectives of the screening by a senior Urologist, the lecture was delivered in Gujarati. On the day of screening the subjects were extensively counseled through dialogue, posters and pamphlets about PSA, lower urinary tract symptoms (LUTS) and carcinoma prostate.

**Figure 1:**
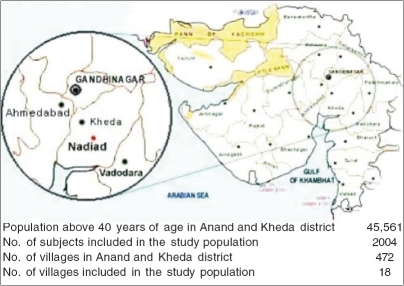
Study population included for the community screening

The study population is not an ethnically distinct group but is representative of the overall population of Gujarat [Figure 1]. The study population comprised adivasis (22%), rural (43%) and urban (35%) subjects. Two thousand four hundred and six subjects attended the community-screening program. All subjects underwent a detailed clinical evaluation that included a digital rectal examination (DRE), uroflowmetry and transabdominal sonography. All the three were done by two Urologists who attended all the screening venues.

International prostate symptom score validated in local language was recorded. Blood was drawn for serum PSA estimation. Subjects requiring any intervention were dealt with as needed. Subjects without any urological ailment were considered healthy. Two thousand and four subjects were evaluable. Due to logistic reasons we randomly selected three villages in which transrectal sonography (TRUS) was done in addition to transabdominal sonography. Patients with abnormal DRE and / or raised PSA and abnormal findings on TRUS underwent further evaluation for prostatic malignancy. A written consent was obtained before subjecting the patient to TRUS biopsy.

All serum PSA estimations were carried out with radiometric assay technique (Diagnostic system Laboratory, Texas, United states). The standard reference range for this assay was 0.0 - 4.0 ng/ml. The assay was conducted at the Central Clinical Laboratory, Department of Pathology, Muljibhai Patel Urological hospital, Nadiad, Gujarat. All the TRUS biopsy cores were seen by a single pathologist at our Central clinical laboratory.

All PSA determinations were made prior to any prostatic manipulations including DRE and TRUS. Transrectal sonography was performed with a 7.5 MHz side-firing endorectal probe (B - K Medical Panther, Advanced Diagnostic Imaging) to assess the prostate volume. The prostate volume was assessed by using the ellipsoid formula.[3,4] PSA density was calculated by dividing serum PSA with prostate volume.[5]

### Statistical analysis

The upper limit of normal for calculating the age-specific PSA values and PSAD was considered as 95^th^ percentile. The value was calculated after sorting the data decade-wise. Correlation between PSA, age and prostate volume was calculated by using Pearson's correlation (two-tailed) coefficient. Correlation coefficients and trend lines were plotted using SPSS version 10.0.

## RESULTS

The mean age of the study population was 62.1 ± 9.5 years. One thousand seven hundred and eighty-seven (89.17%) had a PSA of less the 4 ng/ml,180 (8.9 %) had a PSA between 4-10 ng/ml, while 37 (1.8%) had a PSA more than 10 ng/ml.

All patients with a PSA of more than 4 ng/ml and or abnormal DRE were asked to follow up for further evaluation. Biopsy-proven prostatic malignancy was detected in 20 patients (mean PSA 12.8, range 1.5-40). Among the subjects with positive biopsy seven underwent radical prostatectomy.

**Figure 2:**
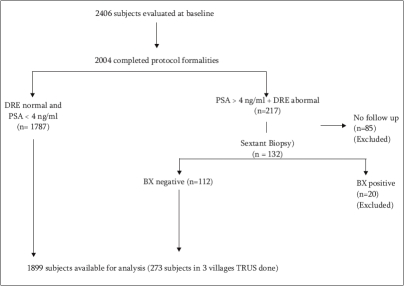
Study design- flow chart

We excluded subjects who did not follow up (n= 85) and who had biopsy-proven malignancy (n=20) from further analysis. One thousand eight hundred and ninety-nine subjects were available for the present analysis [Figure 2].

**Figure 3:**
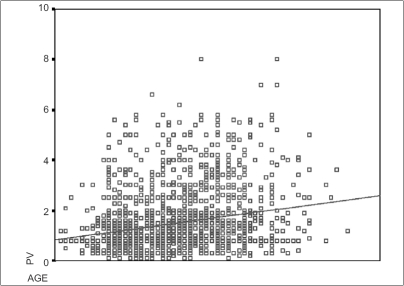
PSA (ng/ml) as a function of age (years)

**Figure 4:**
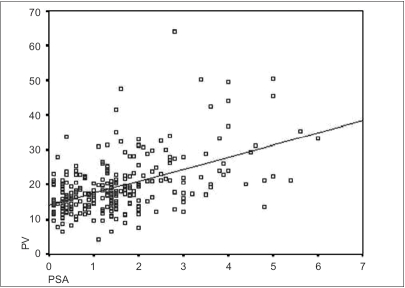
PSA (ng/ml) as a function of prostate volume (ml) (n = 273)

Age-wise distribution of PSA of the entire population is shown in the scattergram [Figure 3] and the values are as shown in Table 1; the serum PSA values linearly increased with age. The distribution of PSA as a function of prostate volume (TRUS volume) is shown in the scattergram [Figure 4].

**Table 1:** Age-specific prostate specific antigen values (ng/ ml) (n = 1899)

Age (years)	Mean + range	95^th^ percentile value
40-49 (n= 124)	1.0 ± 0.6	2.1
50-59 (n= 653)	1.2 ± 1.0	3.4
60-69 (n= 681)	1.5 ± 1.2	4.2
>70 (n= 441)	1.9 ± 1.5	5.0

There was a statistically significant correlation between age and prostate volume (correlation coefficient 0.33); similarly the correlation between prostate volume and PSA was statistically significant, though the value of correlation coefficient of 0.50 may be debatable [Table 2].

**Table 2:** Correlation of prostate specific antigen (ng/ml), Age (years), prostate volume (ml) and prostate specific antigen density (ng/ml/ml) (n = 273)

		PSA	AGE	PV	PSAD
PSA	Pearson correlation	1.000	0.298**	0.503**	0.760**
	Sig. (2-tailed)	-	-	-	-
	N	273	273	273	273
AGE	Pearson correlation	0.298**	1.000	0.332**	0.132*
	Sig. (2-tailed)	0.000	-	-	0.029
	N	273	273	273	273
PV	Pearson correlation	0.503**	0.332**	1.000	-0.061
	Sig. 2 (2 tailed)	-	-	-	0.314
	N	273	273	273	273
PSAD	Pearson correlation	0.760**	0.132*	-0.061	1.000
	Sig. (2 tailed)	-	0.29	0.314	-
	N	273	273	273	273

** Correlation is significant at the 0.01 level (2-tailed).

* Correlation is significant at the 0.05 level (2-tailed), PSA - Prostate specific antigen, PSAD - Prostate specific antigen density

The 95^th^ percentile value for PSAD was 0.19 [Figure 4] in those subjects studied (n=273).

## DISCUSSION

It has been suggested that serum PSA varies among different races.[6] Osterling *et al* have described age-specific PSA reference ranges for the healthy cohort of Japanese men. They compare the PSA as well as the PSAD values of the study population with western literature [Table 3]. Japanese men have lower age-specific PSA and higher PSAD values [Table 3].[2,7] Gupta *et al* noted that Japanese men with LUTS and clinical benign prostatic hyperplasia (BPH) produce and /or release more PSA per unit volume than western men. The apparent difference was attributed to difference in composition of the prostates.[8] An Indian study by Agarwal *et al* describes the PSA and PSAD values in a hospital-based population. The values tend to be higher than those reported in the western literature.[9] Although a study from Andhra Pradesh, India evaluates the causes of LUTS in community-based population,[10] there is a paucity of literature describing the PSA ranges in a community setting. Though the study population in our analysis represents the population in Gujarat, India being a socioculturally and ethnically diverse country, the results cannot be necessarily extrapolated to the whole Indian population. In our study the age-specific PSA values were comparable to the age-matched Japanese population [Table 3], however they differed from the Western literature. Decade-wise, the prostate volumes are also comparable to the Japanese population[2] [Table 3].

**Table 3:** Age-specific and prostate specific antigen density reference ranges for serum prostate specific antigen concentration, Prostate: A comparison with Japanese study and American study

Age range (years)	Serum prostate specific antigen (ng / ml)			
		
	Japanese^2^ (n=286)	Western^7^ (n=471)	MPUH	MPUH
	Osteling *et al*	Osterling *et al*	(n=1899)	Mean + SD
40-49	0.0-2.0	0-2.5	0.0-2.1	1.0 ± 0.6
50-59	0.0-3.0	0-3.5	0.0-3.4	1.2 ±1.0
60-69	0.0-4.0	0-4.5	0.0-4.2	1.5 ± 1.2
> 70	0.0-5.0	0-6.5	0.0-5.0	1.9 ± 1.5
Age range (years)	Prostate volume (ml)			
		
	Japanese^2^	Western^7^ (n=286)	MPUH	Mean
	Osteling *et al*	(n = 471) Osterling *et al*	(n=273)	(MPUH) ± SD
40-49	9-33	13-51	8-21	14.5 ± 3.1
50-59	9-35	15-60	6-42	17.7 ± 5.7
60-69	10-37	17-70	7-45	19.8 ± 7.4
> 70	11-40	20-82	5-64	24.8 ± 12.7
Age range (years)	Prostate specific antigen density (ng/ml/ml)			
		
	Japanese^2^ (n=286)	Western^7^ (n=471)	MPUH	Mean
	Osteling *et al*	Osterling *et al*	(n=273)	(MPUH) ± SD
40-49	0.0-0.10	0.0-0.08	0.0-0.15	0.07 ± 0.05
50-59	0.0-0.12	0.0-0.10	0.0-0.19	0.07 ± 0.05
60-69	0.0-0.15	0.0-0.11	0.0-0.19	0.08 ± 0.05
>70	0.0-0.18	0.0-0.13	0.0-0.20	0.10 ± 0.07

In the recent years there is more widespread support for using a lower PSA threshold in the young population; recent investigations note that biopsy in men with PSA between 2.6 and 4.0 ng/ml may detect clinically significant prostate cancers at an organ-confined stage.[11] The age-specific and PSAD ranges in our study [Table 3] give a suggestion as when to evaluate a subject for malignancy. For example, with a range of 0.0 - 2.1 [Table 1] as suggested in this investigation, a PSA of 2.2 with a PSAD of 0.15 in a 48-year-old Indian man would require further evaluation with TRUS and biopsy.

The prostate volume in our study population was lower as compared to the published literature, however analysis of the relative amount of stromal and glandular tissue contributing to BPH in Indians and the genetic aspect analysis may be of interest. Lower PSA levels in our study population may also perhaps reflect a lower prevalence of prostate cancer in this part of the world.

## CONCLUSION

Indians being ethnically distinct, need to have separate PSA reference ranges which need to be established with large community-based multicentre Indian studies. The age-specific PSA ranges for men in our study population are on the lower side and the PSAD on the higher side. The study also indicates that serum PSA correlates with age, which is primarily due to increasing prostate volume as age advances.
